# Incidence and Treatment of Chylothorax in Children Undergoing Corrective Surgery for Congenital Heart Diseases

**DOI:** 10.21470/1678-9741-2017-0011

**Published:** 2017

**Authors:** Nicolle Martin Christofe, Cristiane Felix Ximenes Pessotti, Laércio Paiva, Ieda Biscegli Jatene

**Affiliations:** 1 Faculdade de Medicina do ABC, Santo André, SP, Brazil.; 2 Hospital do Coração (HCor), São Paulo, SP, Brazil.

**Keywords:** Heart Defects, Congenital, Cardiac Surgical Procedures, Postoperative Complications, Chylothorax

## Abstract

**Introduction:**

Chylothorax is a lymphatic extravasation into pleural cavity and its incidence is 0.25%-5.3% in children undergoing cardiac surgery.

**Objective:**

To evaluate the incidence of chylothorax in pediatrics patients operated, linking it in each surgical intervention. Evaluate treatment types and efficiency.

**Methods:**

Retrospective study using medical records of children undergoing cardiac surgery in the Hospital do Coração between 2004 and 2014. For statistical analysis, qualitative variables by absolute frequency and relative frequency; quantitative variables, by median of 25 and 75 percentiles, as they did not present normal distribution (Shapiro-Wilk, *P*<0.05). The Chi-square test was used for the association between type of treatment and result. The adopted confidence level was 95%.

**Results:**

Incidence of chylothorax was 2.1% (0.9% in intracardiac surgery, 1.7% correction of patent ductus arteriosus and aortic coarctation, 8.3% Glenn's surgery, 11.8% total cavopulmonary surgery and 3% in others). Among treatments, fasting associated with total parenteral nutrition (TPN) resolved 51% of the cases. Hypoglossal diet had failed treatment and surgical referral in 22% of the cases. Fasting with TPN associated with octreotide had success in the treatment of chylothorax in a period exceeding 15 days in 78% of cases, and 3.7% were referred for surgery.

**Conclusion:**

According to the results, incidence of chylothorax was 2.18%. Treatment with fasting and TPN leads to resolutions in 86.5%, and the association with octreotide was successful in 85.1% of cases, showing an efficient option, while the treatment with hypoglossal diet had therapeutic failure in 22% of the cases in which it was used.

**Table:** 

Abbreviations, acronyms & symbols	
TPN	= Total parenteral nutrition

## INTRODUCTION

Chylothorax is a lymphatic fluid extravasation into pleural cavity. In undergoing cardiac surgery children, chylothorax incidence is between 0.25% and 5.3%^[1-6]^ and may occur through direct injury, mainly in the correction of the ductus arteriosus and coarctation of the aorta, due to the anatomical proximity to the thoracic duct originating from the chyle cistern, between L3 and T10, ascends to the thoracic region at the time of aortic hiatus between T10 and T12, and ends its course in an anastomosis at the junction of the subclavian vein and left internal jugular vein^[7]^. It is also frequently related to surgeries for correction of right ventricular outflow obstruction: tetralogy of Fallot and atresia of the pulmonary artery with or without ventricular septal defect.

Chylothorax can also occur due to an increase venous pressure in the superior vena cava, the site of the thoracic duct opening, mainly related to Glenn's operation and total cavopulmonary derivation^[8]^. Recently, with the evolution of pediatric cardiac surgery and concomitant increase in complexity, an increase in the incidence of chylothorax as a postoperative complication of these procedures could be noted, and pediatric cardiac surgery is now the main cause of chylothorax in terciary hospitals^[2,4]^.

Other possible causes of chylothorax are traumatic ones, such as neoplasms and infections of the lymphatic system; and traumatic, such as open traumas^[9]^.

The persistence of chylothorax with continuous lymph extravasation may lead to the depletion of lymphocyte mass, nutrients, proteins, immunoglobulins, coagulation factors, vitamins^[8,10]^, increasing postoperative morbidity and mortality due to infectious, nutritional complications and longer hospital stay.

Regarding the existing treatments for chylothorax, some conservative proposals aim to decrease the production of the pound and its secretion in the pleural cavity. After pleural drainage, one option is a hypoglossal diet, which decreases the intake of long chain triglycerides, preferring the short and medium chains. Another proposal consists of oral fasting with total parenteral nutrition (TPN) for one week and then progressive introduction of food and lipids.

In the last years, new conservative options have been studied that corroborate with the effectiveness of the nutritional restrictions. One option that has gained credibility is the use of octreotide^[11]^ and similar, such as sandostatin^[12]^. Its mechanisms of action are still not clear, but are believed to act in the splenic and hepatic circulation, reducing lymphatic flow through octreotide receptors in this organ, reducing time and optimizing treatment efficacy.

There are also surgical treatments, among them: direct ligation of the thoracic duct, simple raffia of the fistulous area, use of fibrin sealants, pleurodectomy and pleurodesis, and others.

Due to the lack of consensus and in order to minimize the differences between the procedures adopted, Panthongviriyakul and Bines^[13]^ developed an algorithm. This determines the accomplishment of a conservative treatment associated to pleural drainage for two weeks and if chylothorax persisted, then the surgical treatment would be applied^[6,13]^.

Based on the importance of postoperative chylothorax in pediatric cardiac surgery, in order to significantly compromise postoperative morbidity and mortality, we feel stimulated to evaluate the evolution and results of the treatments used in our institution in recent years, seeking to evaluate the treatments used, the changes in these treatments over the years in order to corroborate the search for the best therapeutic strategies to reduce the morbidity of chylothorax in the postoperative period of pediatric cardiac surgery.

### Objectives

Primary

1. Evaluate the incidence of postoperative chylothorax in pediatric cardiac surgery.
2. Evaluate the occurrence of chylothorax associated to each surgical procedure.
3. Evaluate the types of treatment employed with regards to the conservative or invasive strategy.

Secondary

1. Evaluate the success rate of each of the strategies employed.

## METHODS

This is a retrospective study, approved by the Ethics and Research Committee of the Hospital do Coração, through a survey of patient's charts submitted to cardiac surgery for the correction of congenital heart diseases that progressed to postoperative chylothorax, from January 2004 to December 2014.

A descriptive analysis was carried out regarding to:

- Patient age at time of correction
- Gender
- Type of surgery performed
- Post-operative day in which a diagnosis of chylothorax was given
- Technique used for the diagnosis of chylothorax
- Therapeutic strategy employed
- Success in treatment
- Chylothorax treatment time until drain removal

### Inclusion Criteria

Patients undergoing cardiac surgery in the age between 0 and 18 years old for the correction of congenital heart disease who evolved to postoperative chylothorax.

Patients with incomplete or inaccurate data had to be excluded to avoid information breaches.

### Statistical Analysis

The data were recorded in Excel spreadsheet for proper storage.

The statistical analysis had qualitative variables presented by absolute frequency and relative frequency, and quantitative variables by median of 25 and 75 percentiles, as the variables did not present a normal distribution (Shapiro-Wilk, *P*<0.05). To analyze the association between the type of treatment and the result obtained was used the Chi-square test. The confidence level adopted was 95%.

## RESULTS

There were 4099 pediatric patients operated in our service between 2004 and 2014, 87 presented chylothorax, presenting an incidence of 2.12%. Among those studied, 48 (55.1%) were male and 39 (44.9%) were female, 17 (19.54%) had associated Down syndrome, while only 3 (3.45%) had other syndromes. The patients' age at surgery ranged from 0 to 71 months, with a median of 7.83 months.

When analyzing the patients according to the type of surgery performed, cavopulmonary bypass surgery has the highest incidence of chylothorax (11.88%), while intra-cardiac patients have the lowest rates (0.96%) (Table 1).

**Table 1:** Chylothorax incidence by performed surgery.

Surgery type	Number of surgeries with chylothorax/ total surgeries	Chylothorax incidence
Intra-cardiac	24/2485	0.96%
Glenn	14/167	8.36%
Total cavopulmonary	17/143	11.88%
Canal	7/397	1.76%
Aortic coarctation	4/226	1.77%
Others	21/618	3.08%

For the diagnosis of chylothorax, the triglyceride count in the pleural fluid was used, the dosage being considered low from 100 to 200 mg/dl, moderate from 200 to 500 mg/dl and high above 500 mg/dl. Low and moderate dosages were found in equal percentages (41.1%), while high dosages were present in only 17.8% of the studied patients. When we tried to associate the triglyceride level in the pleural fluid at the time of treatment, none significant statistic relation was found (*P*=0.2892).

The day of diagnosis of chylothorax extended from the first to the 51^st^ postoperative day, the median being around the 10^th^ day. In relation to the strategy used for treatment, the Service used these options:

- Hypoglossal diet,
- Fasting (by enteral way) associated with TPN,
- Fasting with TPN associated with the use of octreotide after one week of trying only fasting with TPN or
- Fasting and maintenance of the hypoglossal diet (referred in our hospital as the "Quilothorax Diet 3" for a period of 30 days with outpatient follow-up) (Table 2).

**Table 2:** Used treatment frequency

Treatment	Frequency	Percentage
Hypoglossal diet	9	10.34%
Fasting + TPN	45	51.72%
Fasting + TPN + Octreotide	27	31.03%
Fasting + Chylo III diet	6	6.90%

TPN=total parenteral nutrition

The resolution of the treatment can be summarized in Table 3. The clinical resolution was divided into three periods: 0 to 7 days, 8 to 14 days or more than 15 days. Clinically unresolved cases were referred for surgery and 9.2% evolved to death (Table 3).

**Table 3:** Treatment outcome.

Results	Frequency
Resolved between 0-7 days	6.90%
Resolved between 8-14 days	27.59%
Resolved in more than 15 days	50.57%
Do not resolved, surgery	5.75%
Do not resolved, died	9.20%

The outcome of the treatment was related according to the strategy used (Table 4).

**Table 4:** Association between outcome and used strategy.

Results	Hypoglossal diet	Fasting + TPN	Fasting + TPN + Octreotide	Fasting + Chylo III diet
Resolved in 0-7 days	22.2%	8.8%	__	__
Resolved in 8-14 days	22.2%	42.2%	7.4%	16.6%
Resolved in more than 15 days	33.3%	35.5%	77.7%	66.6%
Do not resolved, surgery	22.2%	2.2%	3.7%	16.6%
Do not resolved, died	__	11.1%	11.1%	__
Total	100%	100%	100%	100%

TPN=total parenteral nutrition

## DISCUSSION

Our study found incidence of chylothorax of 2.12%, rate compatible with the findings in literature such as Mery et al.^[10]^. This same study reveals that the highest incidences of chylothorax present in total cavopulmonary surgeries, similar to that found in our study, in which Glenn also shows a high incidence, only behind of the total cavopulmonary surgeries. Currently, the use of hypoglossal diet as a treatment of chylothorax was abandoned in our Service given preference for fasting and possible associations. In our service, the preference for fasting associated with TPN has increased, and this study shows that 51% of the patients who received this therapeutic option obtained clinical results in less than 14 days. The use of fasting with TPN associated with octreotide also gained space, and it has been administered in more than 30% of the studied patients, reaching resolution rates in 85.1% of cases. In our service we started with a dose of 1 mcg/kg/h in a continuous infusion pump, with a maximum dose of 8 mcg/k/h. Cannizzaro et al.[1] also demonstrated an efficient use of octreotide, reaching resolution levels around 50%. The option to maintain chylothorax III diet in extra-hospital follow-up had its highest rates in patients with clinical resolution above 15 days, which reveals a good general condition for the patient discharge, requiring only follow-up and progression of the diet at home. It is observed that the mortality of patients with chylothorax is high and reaches more than 20% of the studied cases, which highlights the importance of the search for more information and improvement of techniques aimed in solving this complication.

Our study shows that, currently, the best strategy has been the association of fasting with TPN, however, and it should be taken into consideration that the association of octreotide after a week of trying only fasting and TPN has a high resolution index and its use can be considered from the beginning of treatment with the objective to reduce treatment time.

### Limitation

It is a retrospective study, with diversity in the treatments used, which makes it difficult to define the best treatment. A prospective study comparing treatment with fasting with TPN compared to fasting associated with TPN and octreotide is necessary to evaluate whether the use of octreotide interferes with the efficacy of treatment. In addition, it should be noted that in the package inserts of medications such as Sandostatin there is still no formal indication for its use in chylothorax.

## CONCLUSION

The incidence of chylothorax in pediatric cardiac surgery in our service is 2.12%. Chylothorax was more common in the postoperative period of total cavopulmonary bypass (11.8% of the patients submitted to this surgery had chylothorax in the postoperative period), followed by Glenn's operation (8.36%). Among the therapeutic strategies used, fasting with TPN (51.72%) followed by the association of octreotide (31.03%) initiated after treatment failure after one week of fasting. The use of hypoglycemic diet was abandoned in our service. The therapeutic failure occurred in 22.2% of the patients in whom the therapeutic strategy was the hypoglossal diet, 2.2% in cases of fasting with parenteral nutrition and 3.7% in the group in which it was associated with octreotide, evidencing the superiority of fasting in treatment when compared to hypoglossal diet.

**Table:** 

Authors' roles & responsibilities	
NMC	Substantial contributions to the conception or design of the work; or the acquisition; final approval of the version to be published
CFXP	Drafting the work or revising it critically for important intellectual content; final approval of the version to be published
LP	Acquisition, analysis, or interpretation of data for the work; final approval of the version to be published
IBJ	Agreement to be accountable for all aspects of the work in ensuring that questions related to the accuracy or integrity of any part of the work are appropriately investigated and resolved; final approval of the version to be published
